# Avian Primordial Germ Cells Are Bipotent for Male or Female Gametogenesis

**DOI:** 10.3389/fcell.2021.726827

**Published:** 2021-09-29

**Authors:** Maeve Ballantyne, Lorna Taylor, Tuanjun Hu, Dominique Meunier, Sunil Nandi, Adrian Sherman, Brenda Flack, John M. Henshall, Rachel J. Hawken, Mike J. McGrew

**Affiliations:** ^1^The Roslin Institute and Royal (Dick) School of Veterinary Studies, University of Edinburgh, Edinburgh, United Kingdom; ^2^Centre for Tropical Livestock Genetics and Health, University of Edinburgh, Edinburgh, United Kingdom; ^3^Cobb-Europe, Colchester, United Kingdom

**Keywords:** primordial germ cell, avian, chicken, gametogenesis, sex determination

## Abstract

In birds, males are the homogametic sex (ZZ) and females are the heterogametic sex (ZW). Here, we investigate the role of chromosomal sex and germ cell competition on avian germ cell differentiation. We recently developed genetically sterile layer cockerels and hens for use as surrogate hosts for primordial germ cell (PGC) transplantation. Using *in vitro* propagated and cryopreserved PGCs from a pedigree Silkie broiler breed, we now demonstrate that sterile surrogate layer hosts injected with same sex PGCs have normal fertility and produced pure breed Silkie broiler offspring when directly mated to each other in Sire Dam Surrogate mating. We found that female sterile hosts carrying chromosomally male (ZZ) PGCs formed functional oocytes and eggs, which gave rise to 100% male offspring after fertilization. Unexpectedly, we also observed that chromosomally female (ZW) PGCs carried by male sterile hosts formed functional spermatozoa and produced viable offspring. These findings demonstrate that avian PGCs are not sexually restricted for functional gamete formation and provide new insights for the cryopreservation of poultry and other bird species using diploid stage germ cells.

## Introduction

Germ cells from different reproductive stages can be directly transplanted into avian embryos in “windowed” eggs to form germline chimeras in a procedure with minimal welfare implications (reviewed in Panda and McGrew, 2021). The resulting chimeras, or surrogate host birds, will produce sperm or eggs from the donor genetic material when reaching sexual maturity. Chicken primordial germ cells (PGCs) can be isolated from the early embryo [stage 16 Hamburger-Hamilton (HH)] during their migratory stage of development, propagated *in vitro* using a defined serum-free medium and cryopreserved. When re-introduced into surrogate host embryos they will colonize the gonads and form functional gametes in the adult chicken. Cryopreserved diploid PGCs coupled with surrogate hosts promises to be an efficient method for the cryopreservation of avian species (Glover and McGrew, 2012; Nakamura et al., 2013).

During the normal ontogeny of the germ cell lineage, germ cells become restricted to a male or female developmental fate after completing their migration to the bipotential gonad anlage (Adams and McLaren, 2002). Germ cells begin to express ovarian or testes specific genes appropriate to their gonadal environment, which culminates in early embryonic entry into meiosis for female ovarian germ cells and meiotic quiescence for male germ cells until the onset of sexual maturation. The onset of sexual breeding is accompanied by oocyte maturation in females and spermatogenesis in males. In some teleost fish species with genetic sex determination, germ cells from adult gonads will form functional oocytes or spermatozoa in the reverse sex gonads (Okutsu et al., 2006; Dranow et al., 2013). For amphibian and reptilian species, the findings are varied; in species without genetic sex determination, various environmental factors can determine the development of a sexual phenotype with accompanying gamete formation. In species with genetic sex determination, sex-reversed animals have been observed to be fertile in many species, suggesting that in most amphibian and reptilian species germ cells are bipotential in fate and can form either sperm or eggs independent of sex chromosome composition (Wallace et al., 1999; Flament, 2016). In mammalian species, PGCs are restricted in the generation of functional gametes. Germ cell transplantation experiments in mice have shown that chromosomally male (XY) germ cells formed ovarian follicles but these have seldom been observed to produce mature oocytes or offspring (Ford et al., 1975; Eicher et al., 1982). Similarly, chromosomally female (XX) germ cells colonized the male gonads and adult testes, but could not complete meiosis which can be attributed to either the lack of Y chromosome gene products or the presence of two copies of the X chromosome (Palmer and Burgoyne, 1991). These results suggest that sex-restricted gamete differentiation was acquired in mammals during sex chromosome differentiation and vertebrate speciation.

In bird species, males are the homogametic sex and contain two Z sex chromosomes while females are heterogametic and contain a single Z and one W chromosome. Avian PGCs form precociously in the pre-laid egg before the formation of the embryonic germ cells. In chicken, somatic cells were shown to have a cell autonomous sex identity that was independent of gonadally produced sex hormones (Zhao et al., 2010). PGCs at migratory stages also show a sex-specific proteome (M. Govoroun, under review) suggesting that they also have a sex identity. Supporting this hypothesis, germline transmission of *in vitro* propagated donor chicken PGCs was not observed in wildtype or irradiated reverse sex hosts (van de Lavoir et al., 2006; Macdonald et al., 2010). Similarly, genetically sterile *DDX4* knockout female surrogate host chicken carrying *in vitro* propagated donor male PGCs did not contain mature oocytes in their ovaries nor did they lay eggs (Woodcock et al., 2019). In contrasting findings, however, female donor chicken PGCs transplanted into wildtype male surrogate host produced semen PCR positive for the W chromosome (Tagami et al., 1997). However, few W-bearing differentiated spermatozoa were detected (Tagami et al., 2007). Migratory PGCs directly transplanted into partially sterilized opposite sex surrogate host embryos also formed functional gametes and offspring in both male and female reverse sex hosts albeit at extremely low frequencies (Naito et al., 1999). In a similar result, we demonstrated that *in vitro* propagated male layer PGCs formed oocytes and hatched chicks when transplanted into irradiated female layer surrogate hosts (Liu, Chang et al., 2017). Thus, it is not clear if avian PGCs are fully bipotential in fate and can differentiate into functional gametes in avian host gonads of the opposite sex.

We recently generated a surrogate layer chicken model entirely devoid of endogenous germ cells by using genome editing to introduce an inducible iCaspase9 transgene into the DAZL locus. Activation of the transgene using an inert chemical compound induces cell death exclusively in the germ cell lineage (Ballantyne et al., 2021). Using sterile iCaspase9 surrogate host embryos, we directly regenerated two layer breeds of chicken from cryopreserved genome edited PGCs. In those experiments, we mixed equal numbers of male and female PGCs before injection into the iCaspase9 surrogate host embryos. We observed that the offspring were derived from a single male and a single female PGC, which strongly suggested that the male PGCs formed functional gametes in the male host and the female PGCs formed functional gametes in the female host.

Here, we extend our previous findings by investigating the role of chicken breed and avian sex chromosomes on avian gametogenesis. We first demonstrate that a broiler breed of chicken can be directly revived using Sire Dam Surrogate (SDS) mating of iCaspase9 surrogate host layer birds suggesting that chicken breed differences do not influence germ cell differentiation. We also confirm that avian PGCs are not sexually restricted and will form viable gametes and offspring in both sexes.

## Materials and Methods

### Culturing Silkie Broiler Primordial Germ Cells

Fertile Silkie broiler eggs were obtained from the Silkie pedigree flock kept by Cobb-Europe at the Colchester United Kingdom facility. The Silkie flock are maintained under a scheduled feed regime (not *ad libitum*) and egg lay averages 138 eggs per hen. Silkie broiler eggs were incubated at 37.5∘C in a humidified incubator. A 1.0 μl blood sample was collected from the dorsal aorta of individual day 2.5 (stage 16 HH) Silkie embryos. After blood sampling a tissue sample from each embryo was frozen for sexing as determined by W chromosome PCR analysis (Clinton, 1994). The blood sample containing PGCs was cultured in FAOT medium as previously described (Whyte et al., 2015) for 4 weeks until reaching 50,000–100,000 PGCs. PGCs were cryopreserved in Stem-Cellbanker (Amsbio) in 3–4 aliquots until thawed for surrogate host injections. Once thawed PGCs were cultured for 1 week and the sex of the cultured PGCs was confirmed by a W chromosome PCR analysis (Clinton, 1994).

### Generating and Hatching Surrogate Hosts

The iCaspase9 lines of chicken were generated on a Hy-line Brown layer background. A male homozygous iCaspase9 chicken was mated to Hy-line Brown layer hens to produce heterozygote iCaspase9 surrogate host eggs for incubation, injection, followed by hatching. Fertile eggs from the mating were incubated for 62 h to approximately stage 16 HH. A 1.0 μl solution containing 3,000 total male or female cultured PGCs in PGC culture medium minus growth factors and 0.5 mM f.c. B/B compound (Takara Bioscience) was injected into the dorsal aorta through a small window made in the pointy end of the egg. 50 μl of Penicillin/Streptomycin (P/S) solution (containing 15 μM f.c. B/B compound) was pipetted on top of the embryo. The window was sealed with Leukosilk (BSN Medical) and eggs were incubated at 37.5∘C, 45° rock until hatching. Hatchlings were raised until sexual maturity when natural matings in floor pens were set up as indicated. All iCaspase9 and Hy-line birds were fed *ad libitum* and provided with perches and environmental enhancements. All animal management, maintenance and embryo manipulations were carried out under United Kingdom Home Office license and regulations. Experimental protocols and studies were approved by the Roslin Institute Animal Welfare and Ethical Review Board Committee.

### Genotyping

The iCaspase9 transgene was detected using primers TGCCTG GTTGCTTTAATTTCCTC and GGAACAGGTAAAACAGA ACACA, in a PCR reaction of 95∘C/5 min, (95∘C/30 s, 64∘C/30 s, and 72∘C/60 s) x35 cycles, 72∘C/5 min using FastStart Taq DNA polymerase (Merck). A GAPDH control PCR using primers CAGATCAGTTTCTATCAGC and TGTGACT TCAATGGTGACA, and a W chromosome sex PCR using primers GAAATGAATTATTTTCTGGCGAC and CCCAAATA TAACACGCTTCACT were carried out in a PCR reaction of 94∘C/4 min, (94∘C/30 s, 60∘C/30 s, and 72∘C/30 s) x35 cycles, 60∘C/10 min. PCR to simultaneously detect the Z and W chromosomes was carried out using primers GAGA AACTGTGCAAAACAG and TCCAGAATATCTTCTGCTCC in a PCR reaction of 95∘C/5 min (95∘C/30 s, 50∘C/30 s, and 72∘C/30 s) x 40 cycles, 72∘C/5 min (Kahn et al., 1998).

### Principal Component Analysis

Genomic DNA from G_1_ offspring and control birds [Hy-line Brown, iCaspase9 (bred on a Hy-line Brown background), and Silkie broiler birds] were genotyped using a custom Cobb 60K Infinium Illumina array. Genomic DNA was prepared from blood from G_1_ chicks using cell lysis solution (Qiagen) containing RNAse A Solution (Merck). In some instances, extra embryonic chorioallantoic membrane samples were used instead of blood and genomic DNA was extracted using REDExtract-N-Amp Tissue PCR Kit (Merck) and cleaned up using Qiagen MinElute PCR purification columns.

## Results

### Biobanking of Broiler Primordial Germ Cells and Hatching of iCaspase9 Sterile Host Chicken

We obtained fertile eggs from a commercial pedigree flock of Silkie broiler chicken as starting material for PGC propagation and cryopreservation. The Silkie broiler chicken has white feathers and dermal hyperpigmentation resulting in black skin and muscle (Bateson and Punnett, 1911). Embryonic blood was sampled from individual day 2.5 (stage 16 HH) Silkie broiler embryos and PGCs were propagated from the embryonic blood in serum-free culture conditions (Whyte et al., 2015). 27 eggs were sampled and 13 PGC cultures were successfully obtained, yielding 6 male and 7 female PGC lines; these were propagated *in vitro* for 3–4 weeks before being cryopreserved in aliquots of 50,000 cells for future transplantation experiments (Supplementary Table 1). Cryopreserved PGCs from two individual male or female lines were thawed after a minimum of 6 month cryopreservation and cultured for several days before being injected into iCaspase9 surrogate host embryos. The surrogate host embryos contain an inducible Caspase9 transgene which is specifically expressed in the germ cells of the developing embryo. Addition of the inert chemical compound, B/B (AP20187), induces dimerization of the iCaspase9 molecule and initiates an apoptotic cascade causing apoptosis of the germ cell lineage (Ballantyne et al., 2021). We injected either an equal number of male or female PGCs from two individual PGC cultures, or PGCs from a single female culture into the iCaspase9 surrogate host embryos at day 2.5 (stage 16 HH). The injected embryos were sealed, incubated to hatching and the hatchlings were raised to sexual maturity before mating to produce chicks for visual analysis and genotyping (Supplementary Table 2). Host embryos were not sex genotyped before injections so that on average 50% of the surrogate hosts were the reverse sex of the donor PGCs.

### Germline Transmission From Surrogate Hosts

**FIGURE 1:**
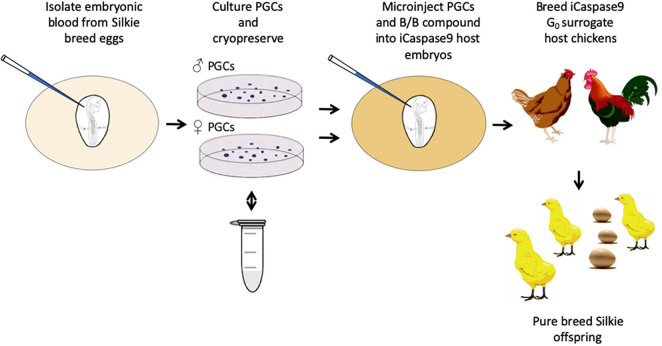
Regeneration of pure breed Silkie chicks using cryopreserved PGCs and sterile iCaspase9 surrogate host embryos.

**FIGURE 2:**
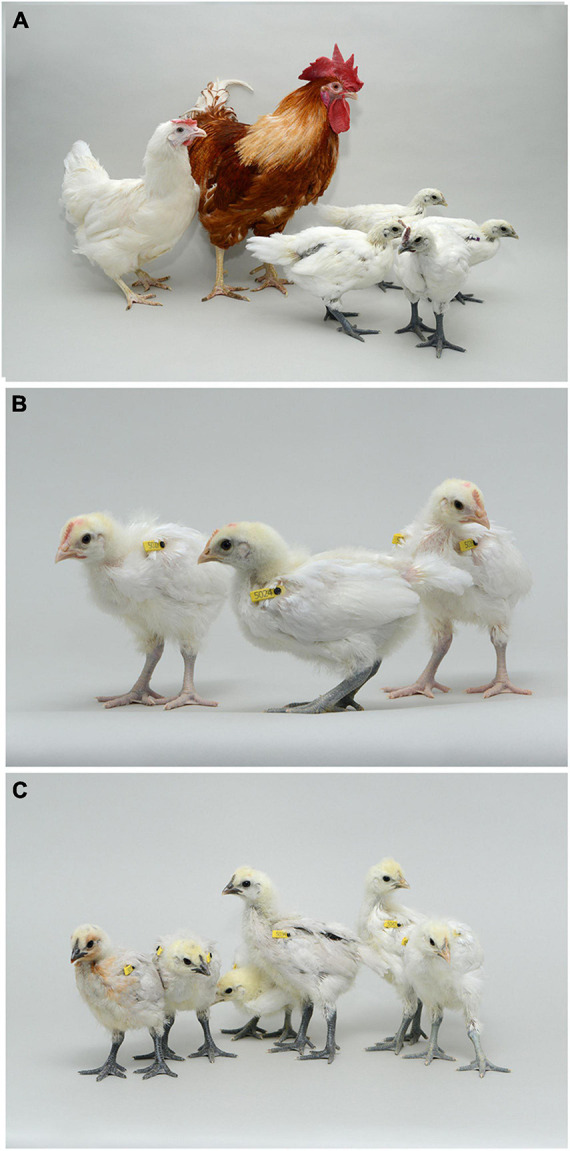
Offspring from iCaspase9 surrogate host matings. **(A)** Offspring displaying black skin (right) derived from an iCaspase9 surrogate host cockerel carrying male Silkie broiler PGCs mated to iCaspase9 surrogate host hens carrying female Silkie broiler PGCs (parents on left). **(B)** Offspring obtained from iCaspase9 surrogate hens carrying male Silkie broiler PGCs mated to a male brown layer cockerel. **(C)** Offspring from iCaspase9 surrogate host cockerels carrying female Silkie broiler PGCs mated to brown layer hens.

**FIGURE 3:**
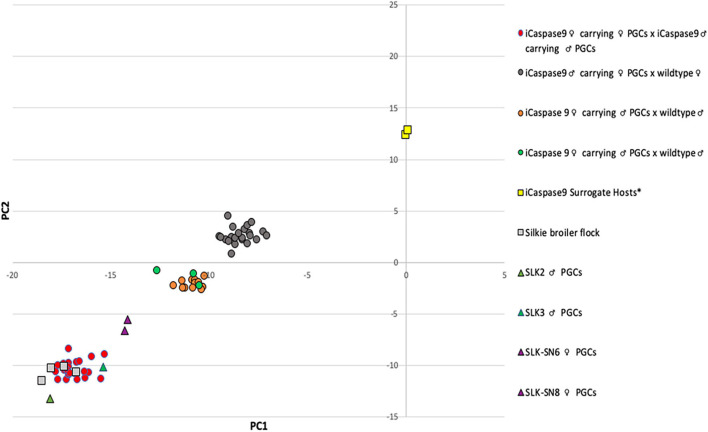
Principal Component Analysis of iCaspase9 surrogate host offspring. PC analysis of genomic DNA from offspring of iCaspase9 surrogate host birds. G_1_ offspring are indicated by circles, control birds are indicated by squares and PGCs are indicated by triangles. *iCaspase9 surrogate hosts are the same genotype as the wildtype brown layer birds.

An iCaspase9 host cockerel, hatched from an embryo injected with two mixed male Silkie PGC lines, was naturally mated to two iCaspase9 host hens, both from embryos injected with two mixed female Silkie PGC lines (SDS mating; Figure 1). These iCaspase9 hens laid 4.84 eggs per hen per week (Table 1, Group 1). In comparison, control layer females laid 6.55 eggs per hen per week (Table 1, Group 2). Eggs from this mating were incubated; fertility was 64%, and 44% of the fertile eggs successfully hatched. The chicks all displayed white feathers and black skin typical of a Silkie broiler (Figure 2A) and no health or behavior abnormalities were observed. PCR analysis of the offspring did not detect the iCaspase9 transgene, suggesting the offspring did not derive from the endogenous germ cells of the surrogate host parents. A Principal Component (PC) analysis using approximately ∼2,000 autosomal single nucleotide polymorphisms (SNP) genotypes from each offspring and parental PGCs, generated from an Illumina 60K Infinium chip was completed. These data demonstrate that the offspring clustered with pure breed Silkie individuals from the source population, indicating that the surrogate offspring were all derived from male and female Silkie donor PGCs (Figure 3).

**TABLE 1:** Offspring from surrogate host birds.

**iCaspase9 host mating groups**	**Individuals in each mating pen**	No. of eggs laid per hen per week^*^	**No. of eggs incubated**	**Fertility^†^ (% eggs incubated)**	**No. of chicks hatched^₴^ (% fertile eggs)**	**^$^ iCaspase9 offspring (%)**
**Group 1**						
♂ surrogates carrying ♂ Silkie PGCs x ♀ surrogates carrying ♀ Silkie PGCs	G_0_SLK9-18♂ x G_0_SLK11-13♀ G_0_SLK11-14♀	4.84	53	34 (64%)	15 (44%)	0
**Group 2**						
♀ surrogates carrying ♂ Silkie PGCs x ♂ control	1 layer cockerel♂ x G_0_SLK6-34♀	3.15	21	13 (62%)	11 (85%)	0
	1 layer cockerel♂ x G_0_SLK9-13♀ G_0_SLK9-19♀	1.98	17	16 (94%)	13 (81%)	0
	**Mean**	2.57	38	29 (76%)	24 (83%)	
**Group 3**						
♂ surrogates carrying ♀ Silkie PGCs x ♀ control	G_0_SLK10-2♂ G_0_SLK10-7♂ x 5 layer hens♀	6.55	109	103 (95%)	43 (33%)	0
	G_0_SLK10-1♂ G_0_SLK10-14♂ x 5 layer hens♀	NA	92	0		

*Lay rate; eggs were counted over a 60 day period when hens were between 7 and 12 months of age and divided by the number of fertile hens present in pen. The maximum possible lay rate is 7.0 eggs per week.

*Group 1 females laid a total of 180 eggs per hen.*

*Group 2 females laid a total of 98 eggs per hen.*

*^†^Fertility; no. of eggs with embryos at day 18 of incubation detected by candling.*

*^₴^Hatchability; no. of chicks hatched from fertile day 18 eggs.*

*^$^No. of offspring PCR^+^ for iCaspase9 transgene.*

The iCaspase9 host hens derived from female embryos injected with Silkie donor male PGCs began laying eggs when reaching sexual maturity at 20 weeks of age. The iCaspase9 hens laid an average of 2.57 eggs per hen per week (Table 1, Group 2). These hens were naturally mated to a control brown layer cockerel and the eggs from the mating were incubated to hatch. Fertility of the incubated eggs was 76%, and 83% of the fertile eggs hatched successfully. Chicks from this mating had black or gray skin with white feathers and were healthy (Figure 2B). All chicks hatched from this mating were male as would be expected for chicks arising from a ZZ × ZZ mating. PCR analysis of the offspring did not detect the iCaspase9 transgene, carried by the surrogate host parents. PC analysis using offspring and parental population genotypes found that the offspring clustered between pure Silkie and control brown layer birds, indicating that the chicks were hybrids between these two breeds of chicken (Figure 3).

In a separate experiment we injected PGCs from a single female Silkie PGC line into iCaspase9 embryos. The iCaspase9 host cockerels derived from these injections were naturally mated to control brown layer hens. Eggs from this mating were incubated to hatch. Fertility from a first pair of cockerels was 0% suggesting that the males did not mate with the females or that they were infertile (Table 1, Group 3). Fertility of the incubated eggs from a second pair of iCaspase9 cockerels was 95% and hatchability of the fertile eggs was 33%. Hatchlings from the mating had black skin with white feathers and were healthy (Figure 2C). A PCR analysis of the offspring did not detect the iCaspase9 transgene carried by the surrogate hosts parents, and subsequent genotype PC analysis showed that the offspring clustered between pure Silkie and control brown layer birds, indicating that the chicks were hybrids between these two breeds of chicken (Figure 3). These results indicate that broiler PGCs carried in layer hosts depleted of endogenous germ cells form functional gametes and pure offspring in SDS matings, and that avian germ cells are not sexually determined and can form functional gametes in reverse sex hosts in the absence of competition from the endogenous germ cells.

### Transmission of Multiple Genotypes From a Single Host Bird

We carried out a pedigree analysis using individual bird genotypes to measure the transmission rate of individual donor genotypes from surrogate host birds carrying two genotypes and to verify the parentage of the offspring (Table 2 and Supplementary Table 3). The two female iCaspase9 hosts carrying two mixed female PGC lines (Group 1) transmitted 11/26 of one genotype (SN6) and 15/26 of the second genotype (SN8) to offspring. In contrast, the two male iCaspase9 hosts carrying two mixed male lines (Group 1) transmitted 23/26 of one genotype (SLK2) and 3/26 of the second genotype (SLK3) to their offspring. This pedigree analysis also confirmed that all offspring were derived from an egg formed from a single male and a single female donor haplotype. Similarly, in female surrogate hosts injected with male donor PGCs (Group 2), 14/14 of the dominant genotype (SLK2) was transmitted to the offspring. The male surrogate hosts injected with the single female genotype (SN6) confirmed that this genotype was transmitted to all offspring (22/22). These data suggest that the male gonadal competition is more intense or that the culturing process may influence germ line transmission of male PGCs more than female PGCs.

**TABLE 2:** Sex and genotype of Silkie offspring.

**iCaspase9 host mating groups**	**Individuals in each mating pen**	**PGC line injected into G_0_ at embryonic stage 16 HH**	**No. of genotyped offspring**	**Sex of offspring**	**Genotype of offspring**
**Group 1**					
♂ surrogates carrying ♂ PGCs x ♀ surrogates carrying ♀ PGCs	G_0_SLK9-18♂ x G_0_SLK11-13♀ G_0_SLK11-14♀	SLK2♂ SLK3♂ ————– SLK-SN6♀ SLK-SN8♀	26	13♂ 13♀	23 SLK2 3 SLK3 11 SLK-SN6 15 SLK-SN8
**Group 2**					
♀ surrogates carrying ♂ PGCs	1 layer cockerel♂ x G_0_SLK6-34♀	SLK2♂	3	3♂	3 SLK2
	1 layer cockerel♂ x G_0_SLK9-13♀ G_0_SLK9-19♀	SLK3♂	12	12♂	12 SLK2
**Group 3**					
♂ surrogates carrying ♀ PGCs	G_0_SLK10-2♂ G_0_SLK10-7♂ x 5 female layer hens♀	SLK SN6♀	22	7♂ 15♀	22 SLK SN6

### Sex Chromosome Analysis of Offspring

We next examined the SNP genotyping data to verify the presence of the sex chromosomes in the offspring from the three surrogate host mating groups (Supplementary Table 3). Surrogate host offspring were initially designated as male or female according to the detection of the W chromosome by PCR analysis. We observed that all putative male offspring – offspring PCR negative for the W chromosome – were heterozygous for Z chromosome SNPs. This result indicates that all putative male offspring contained two Z chromosomes and were not ZO. The putative female offspring – offspring PCR positive for a W chromosome – carried all 11 W chromosome SNP markers and also carried a single Z chromosome haplotype, indicating that all putative female offspring carried a W chromosome and a single Z chromosome. To be noted, 10–12 W chromosomal SNPs were identified in the female Silkie donor PGCs (SLK-SN6 PGCs, SLK-SN8 PGCs) carried by the surrogate hosts, the 1–3 remaining SNPs could not be unambiguously identified (Supplementary Table 3 and Supplementary Figure 1).

Offspring from the females carrying male PGCs (Group 2) lacked all 13 W chromosome markers confirming they derived from a ZZ × ZZ mating.

The offspring from male surrogate hosts carrying female PGCs (Group 3) all carried the Z chromosome haplotype carried by SN6 PGCs (haplotype 13) and female offspring from this mating only contained a single “h13” Z chromosome haplotype. These results indicate that the female PGCs carried in male hosts only transmitted the Z chromosome to offspring. This is a similar result to that observed when female chicken blastodermal cells were transplanted into male recipient embryos (Kagami et al., 1995). Genotyping analysis of embryos at day 3 of incubation (stage 17 HH) from this mating did not detect the presence of WW embryos (see section “Materials and Methods”) and no female hatchlings were heterozygous for the W chromosomal SNPs, adding additional proof that functional W spermatozoa were not formed. Overall, these results demonstrate that female ZW PGCs in the male testes differentiated into functional spermatozoa which transmitted the Z chromosome through the haploid gamete and further confirms that avian PGCs are truly bipotential for forming both oocytes and spermatozoa.

## Discussion

Chickens are the most populous livestock species and are an essential source of protein for human nutrition worldwide (FAOSTAT, 2019). Reproductive technologies play an important part in livestock breeding programs and conservation of genetic diversity. The reproductive germ cells carry the genetic material that is transferred between generations, and cryopreservation of this reproductive material is central to the efficient management and safeguarding of animal genetic resources (Whyte et al., 2016). In avian species, the large yolk filled egg is not amenable to cryopreservation. Likewise, cryopreserved avian spermatozoa exhibit lower fertility in comparison to mammalian species, which can be attributed to the extensive migration and prolonged storage of semen in the avian oviduct (Blesbois et al., 2007). In contrast to mammalian species, the development of the avian embryo in a shelled egg makes it an ideal model for the generation of reproductive cell chimeras. Here, we present a further demonstration of surrogate host technology and *in vitro* propagated PGCs for the “biobanking” of diverse poultry breeds. In this case a commercial broiler line of chicken was directly re-established from cryopreserved reproductive material using layer surrogate host chicken. We showed that the multiplexing of multiple genotypes through surrogate hosts is possible, and that an equal number of offspring was produced from two mixed female genotypes. In contrast, offspring from surrogate hosts carrying mixed male PGCs were skewed toward a dominant “genotype.” Indeed, one of the male PGC lines almost entirely outcompeted the second male line in both the testes and the ovary of iCaspase9 hosts. Future work will determine at what stage this skewing occurs. Potentially, multiplexing additional genotypes in a single host could limit the ability of a single genotype to dominate the gonadal germ cell niche. Also, the elimination of the requirement for the *in vitro* amplification of PGCs before cryopreservation may prevent the selection of a dominant PGC line which may occur during *in vitro* cell culture. A multiplexing step will be necessary to increase the genetic diversity of the offspring obtained from individual surrogate hosts for future efforts to produce an outbred flock from cryopreserved cells.

Broiler chicken pedigree lines experience multiple ovulations, internal laying, and production of soft-shelled and double-yolked eggs when fed *ab libitum* (Hocking and Robertson, 2000). A restricted diet of broiler lines reduces the incidence of multiple ovulations and improves egg quality. Avian ovulation is coordinated by the hypothalamus-pituitary-gonad axis (Sharp et al., 1984; Bédécarrats et al., 2016). It is formally possible that the genetics of the broiler germ cells are responsible for the multiple ovulations and internal laying, however, we found that there was no indication of these complications in the layer host females carrying male or female broiler PGCs. This result indicates that broiler germ cells are not causative for the multiple ovulations that occur in broiler dam lines fed *ad libitum.*

In our previous research, we reported that male broiler PGCs cells could not form functional oocytes in hens genetically modified to lack endogenous germ cells. We found that *DDX4* null female layer hosts injected with female broiler PGCs laid eggs and produced broiler offspring. However, *DDX4* null female layer hosts injected with male broiler PGCs did not ovulate or lay eggs, suggesting that male broiler germ cells could not form viable follicles in female layer hosts (Woodcock et al., 2019). In the *DDX4* null female host, the endogenous PGCs that are present in the embryo are lost between day 3 and day 14 post hatch (Taylor et al., 2017). We hypothesize that the elimination of all endogenous germ cells at early embryonic stages in the iCaspase9 host permitted the donor male broiler PGCs to populate the ovarian niche and form functional follicles. This result also suggests that male germ cells are not as competent as female germ cells to form a functional oocyte in the ovary in the presence of competing female germ cells. Moreover, these data also indicate that the W chromosome is therefore not required by the avian germ cell to form a mature functional follicle. However, we noted that egg production from the female hosts injected with male PGCs was reduced in comparison to female hosts carrying female PGCs (Table 1). The W chromosome could therefore play a role in the proliferation and maturation of the follicle in the ovarian milieu. The lack of global dosage compensation of the Z chromosome and the presence of two ZZ chromosomes in the male PGCs may also interfere with the process of oogenesis (Ellegren et al., 2007; Melamed and Arnold, 2007; Itoh et al., 2010). It was shown that the proteome of male and female PGCs differ especially for genes located on the Z chromosome (M. Govoroun, under review).

As avian female germ cells contain a Z chromosome it would be expected that they, unlike mammalian female (XX) germ cells, contain all gene transcripts cell-autonomously required to form functional spermatozoa. However, in the presence of any male competing germ cells, female germ cells have not been observed to produce functional gametes and offspring, suggesting that spermatogenesis is a highly competitive process (Ramm et al., 2014). Thus, the presence of a W chromosome or possessing a single Z chromosome must limit the female germ cell’s ability to compete for the male gonadal niche. Previous research on meiotic pairing of sex chromosomes in mammals detected clear differences in inactivation of XY chromosomal pairs which occurs during meiosis in the male testes and the lack of silencing of XX chromosomal pairs in the developing female oocyte (Turner, 2007). In chicken, the ZW pair was often observed to avoid synapse formation and inactivation during oocyte meiosis (Guioli et al., 2012). An absence of sex chromosome silencing in the avian species may permit both ZZ oogenesis and ZW spermatogenesis to successfully proceed in reverse sex gonads as observed in this work. In future research it will be informative to observe the motility of the ZW germ cell-derived spermatozoa to determine if they are less motile than ZZ germ cell-derived spermatozoa.

## Data Availability Statement

The original contributions presented in the study are included in the article/Supplementary Material, further inquiries can be directed to the corresponding author/s.

## Ethics Statement

The animal study was reviewed and approved by Roslin Institute Animal Welfare and Ethical Review Board Committee.

## Author Contributions

MB, TH, DM, MM, and AS carried out the embryology. RH, BF, and JH carried out and analyzed the genomic data. SN, MM, and LT performed cell culture. LT and MB genotyped the chickens. All authors analyzed the data and wrote the manuscript.

## Conflict of Interest

BF, JH, and RH were employed by the company Cobb-Europe. The remaining authors declare that the research was conducted in the absence of any commercial or financial relationships that could be construed as a potential conflict of interest.

## Publisher’s Note

All claims expressed in this article are solely those of the authors and do not necessarily represent those of their affiliated organizations, or those of the publisher, the editors and the reviewers. Any product that may be evaluated in this article, or claim that may be made by its manufacturer, is not guaranteed or endorsed by the publisher.
