# Supplementary material for: Avian Primordial Germ Cells Are Bipotent for Male or Female Gametogenesis
Source: Front Cell Dev Biol. 2021 Sep 29;9:726827. doi: 10.3389/fcell.2021.726827 (PMC8511492; doi:10.3389/fcell.2021.726827)
Supplement: Supplementary file 1 [file Data_Sheet_1.docx]

Supplementary Material

**Supplementary Table 1** Derivation of cultured PGCs from Silkie broiler eggs

| Experimental number | Female PGC cultures  (% eggs sampled) | Male PGC cultures  (% eggs sampled) | Total |
| --- | --- | --- | --- |
| 3 | 7/16 (44) | 6/11 (55) | 13/27 (48%) |

Eggs were individually sampled for embryonic blood at stages 15-16^+^ HH. Cultures containing more than 40,000 PGCs at 21 days were scored positive and cultured for an additional week before cryopreservation.

**Supplementary Table 2** Hatching of surrogate hosts

| Injection set | Sex of donor cells | PGC line | No. of embryos injected^£^ | Surrogate host eggs | Day 14 fertility (% injected embryos) | No. & sex of hatchling  (% hatch rate) ^*^ |
| --- | --- | --- | --- | --- | --- | --- |
| SLK6 | M | SLK2, SLK3 | 7 | iC9 het | 5/7 (71%) | 1♂ (20%) |
| SLK9 | M | SLK2, SLK3 | 10 | iC9 HL | 9/10 (90%) | 3♂ + 2♂ (56%) |
| SLK10 | F | SN6 | 25 | iC9, iC9 HL | 18/25 (72%) | 6♂ (33%) |
| SLK11 | F | SN6, SN8 | 22 | iC9 HL | 17/22 (77%) | 2♂ + 6♂ (47%) |

^£^3000-4000 PGCs were injected per surrogate host egg.

*Hatchability; no. of chicks hatched from fertile day 14 eggs.

iC9 het = iCaspase9 heterozygote parental cockerel

iC9 HL = iCaspase9 homozygote parental cockerel


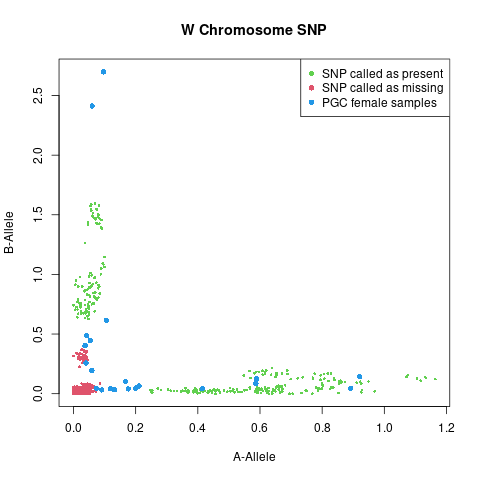


**Supplementary Figure 1** W chromosomal SNPs in the donor female Silkie PGCs

Analysis of the W SNPs in the female PGCs (blue) and female (green) and male (red) surrogate host offspring. Three W chromosomal SNPs were called as missing in the donor female Silkie PGCs.
